# Clinical Characteristics of SARS-CoV-2 Omicron Cases in Pune, Maharashtra, India

**DOI:** 10.7759/cureus.37032

**Published:** 2023-04-02

**Authors:** Rohidas Borse, Rajesh P Karyakarte, Rashmita Das, Sushma Yanamandra, Sonali Salvi, Harshal Bhitkar, Sanjay Mundhe, Dhananjay Ogale, Nagnath Radewad, Suvarna Joshi, Krishanpal Karmodiya

**Affiliations:** 1 Internal Medicine, Byramjee Jeejeebhoy Government Medical College & Sassoon General Hospitals, Pune, IND; 2 Microbiology, Byramjee Jeejeebhoy Government Medical College & Sassoon General Hospitals, Pune, IND; 3 Department of Biology, Indian Institute of Science Education and Research, Pune, IND

**Keywords:** variant of concern, clinical and laboratory characteristics, covid-19, omicron variant, sars-cov-2

## Abstract

Background

The SARS-CoV-2 Omicron variant, within two months of its detection, replaced the Delta variant to become the dominant circulating variant globally. Therefore, it is essential to understand the characteristics of the disease caused by the variant and its impact on vaccination.

Methods

A total of 165 confirmed Omicron cases attending a tertiary care hospital in Pune, Maharashtra, between December 2021 to February 2022 were studied. Their demographic, clinical, and immunization history was recorded.

Results

Among the 165 cases, 7.88% were B.1.1.529 Omicron cases, 25.45% were BA.1 Omicron cases, and 66.67% were BA.2 Omicron cases. Of these 165 patients, 146 (88.48%) were discharged after treatment, 12 (7.27%) died during hospitalization, and seven (4.24%) were brought dead. The presence of one or more comorbid conditions was seen in 15.15%, of which diabetes mellitus and hypertension (28% each) were the most common conditions. Older age (greater than 60 years), an important risk factor for poor outcomes, was present in 9.1% of cases. Among the 165 cases, vaccination with at least one dose of vaccine was found in 80.61% of cases. Out of 165 cases, clinical data was available for 158 cases. Of these 158 cases, 86.71% had symptoms, and 13.29% were asymptomatic. Fever, followed by cough, myalgia, runny nose, and headache, were the most common presenting symptoms. The mean duration of illness was 2.69 days, with 91.14% of cases having the illness for less than five days, and 89.24% of cases had a National Early Warning Score (NEWS) of 1-4, suggesting a good prognosis. In 93.90% of cases, the chest X-ray findings were normal. Of the 158 cases, 92.41% of cases recovered with supportive treatment, and only 7.59% of cases required oxygen therapy.

Conclusion

The current study shows that the Omicron variant caused mild disease with reduced need for hospital admission and oxygen therapy in India.

## Introduction

On 24th November 2021, a novel SARS-CoV-2 variant was first reported from South Africa [1]. The variant was initially identified as Pango lineage B.1.1.529 and was designated as Omicron, a Variant of Concern (VOC) by the World Health Organization on 26th November 2021. This highly mutated SARS-CoV-2 variant had nearly 50 mutations in its genome compared to the original SARS-CoV-2 virus isolated in Wuhan, China. Of these mutations, around 26-30 mutations are of particular interest as they are in the spike protein region, of which 15 were in its receptor-binding domain (RBD) [2]. Since its first identification, the variant has spread rapidly worldwide and accounts for 99% of sequences reported globally [3].

Omicron is a highly transmissible variant with an effective reproduction number 3.19 (95% CI 2.82-3.61) times greater than Delta's. This property accounts for its rapid increase in numbers and higher transmissibility, thus displacing the prevailing Delta variant [4]. Various early studies have shown that extensive immune escape and reduced vaccine effectiveness of the Omicron variant have led to its higher transmission rate. Preliminary studies suggest that the variant causes less severe disease with milder clinical manifestations such as rhinorrhea, sore throat, and headache. Though infections due to the Omicron variant have surged worldwide, it has not led to increased hospitalization in the same way as in the earlier Delta wave [5]. Many demographic studies with evidence of reduced disease severity and hospitalization due to the Omicron variant have been shared by countries with diverse levels of population immunity.

India saw an upsurge in coronavirus disease (COVID-19) cases driven by the Omicron variant in the last week of December 2021 [6]. Omicron emerged during the period when the country already had 57% of the population vaccinated with at least one vaccine dose [7]. In the context of the above-mentioned scenario, the present study describes the epidemiological, clinical characteristics, and outcomes of Omicron-confirmed cases in a tertiary care hospital in Pune, Maharashtra, India. 

## Materials and methods

This clinical study was carried out at the Department of Medicine in collaboration with the Department of Microbiology at Byramjee Jeejeebhoy Government Medical College (BJGMC), Pune, Maharashtra, India. The study falls within the research activities approved by the Institutional Ethics Committee, BJGMC, Pune, Maharashtra (BJGMC/IEC/Pharmac/ND-Dept 0721233-233, dated 15-09-2021).

Sample acquisition for SARS-CoV-2 whole genome sequencing

RT-PCR-positive nasopharyngeal samples of patients attending Sassoon Hospital between December 2021 and February 2022 were collected in a viral transport medium (VTM). The samples were transported to the sequencing laboratory in the Department of Microbiology, BJGMC, Pune, maintaining a cold chain between 2-8^o^C and were stored at -80^o^C until further processing. Samples with a cycle threshold value (Ct value) less than 25 were processed for SARS-CoV-2 whole-genome sequencing.

The extracted RNA samples were sent to the Indian Institute of Science Education and Research (IISER), Pune, and the Centre of Excellence for Genomics, Department of Microbiology, BJGMC for SARS-CoV-2 whole-genome sequencing and lineage analysis.

Library preparation, next-generation sequencings, and lineage analysis

Libraries for COVID-19 were prepared using the defined protocol for Illumina COVIDseq RUO test kits (Illumina Inc., San Diego, CA) at IISER, Pune, and Rapid barcoding and Midnight RT-PCR Expansion kits (Midnight Protocol, Oxford Nanopore Technologies, Littlemore, United Kingdom) at BJGMC. Sequencing was performed using the NextSeq 500 sequencer (Illumina Inc.) at IISR and GridION sequencer (Oxford Nanopore Technologies) at BJCMC. Lineage identification and clade analysis were performed using Pangolin (https://github.com/hCoV-2019/pangolin) and Nextclade software (https://clades.nextstrain.org/).

Data collection and analysis

Demographic characteristics and the clinical findings of Omicron cases were collected from medical and laboratory records maintained by the medical record section of the hospital. The data were entered in Microsoft Excel, and analysis was done using the JMP statistical software, version 13.0.0 (SAS Institute, Cary, NC). A p-value of less than 0.05 (typically ≤ 0.05) was considered a statistically significant result.

## Results

**Figure 1:**
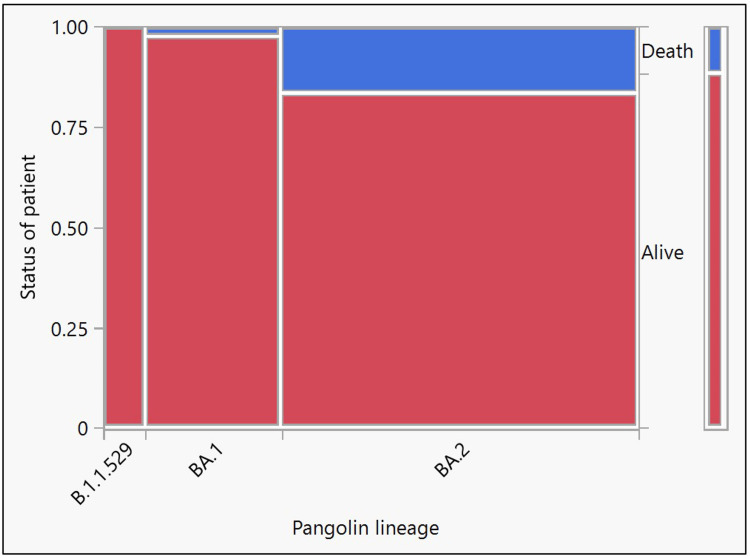
Mosaic plot showing Pangolin lineage versus the outcome of confirmed Omicron cases

A total of 165 confirmed Omicron cases were studied between December 2021 and February 2022. Among the 165 cases, 7.88% were B.1.1.529, 25.45% were BA.1, and 66.67% were BA.2 cases. Of these 165 cases, 146 (88.48%) were discharged after treatment, 12 (7.27%) died during hospitalization, and seven (4.24%) were brought in dead. Figure 1 and Table 1 show the relationship between the Omicron variants and the outcome of the disease. Among the 19 patients who died, one (5.26%) had a BA.1 infection, and 18 (94.74%) had a BA.2 infection (seven (38.89%) were brought in dead, and 11 (61.11%) died during hospitalization).

**Table 1:** Chi-square test results for goodness of fit showing Pangolin lineage versus the outcome of confirmed Omicron cases

Test	ChiSquare (χ2)	Prob>ChiSq (p-value)
Likelihood Ratio	10.37	0.0056
Pearson	7.67	0.0216

Demographic characteristics of COVID-19 cases infected with the Omicron variant of SARS-CoV-2

**Figure 2:**
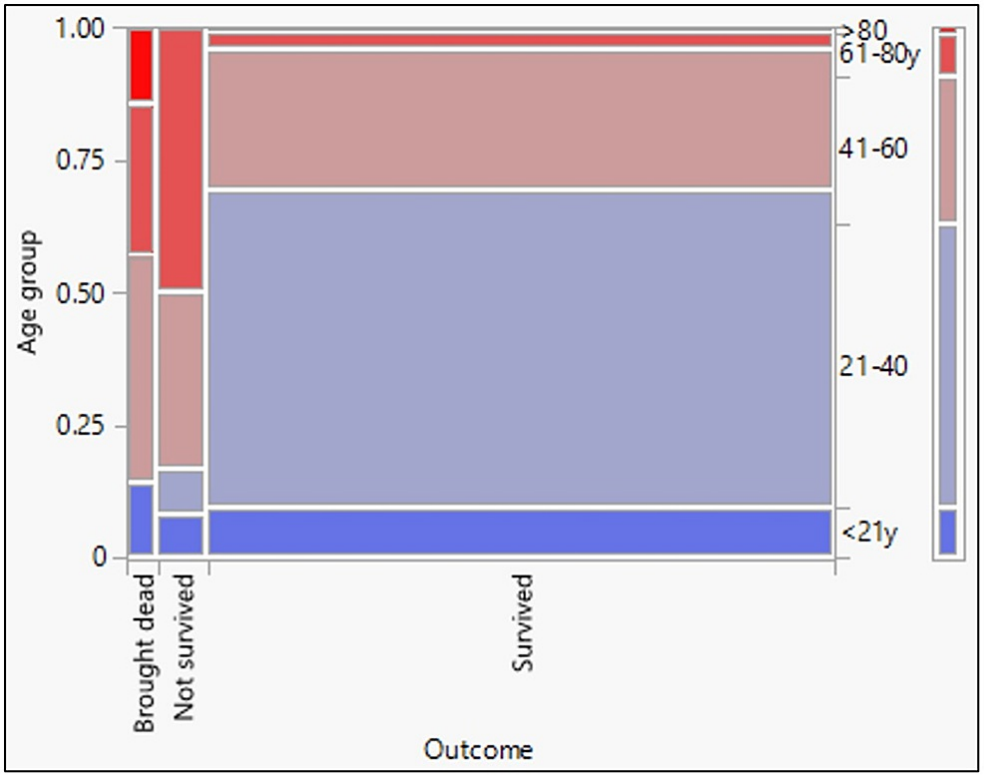
Mosaic plot showing the relationship between age and the outcome of confirmed Omicron cases

**Figure 3:**
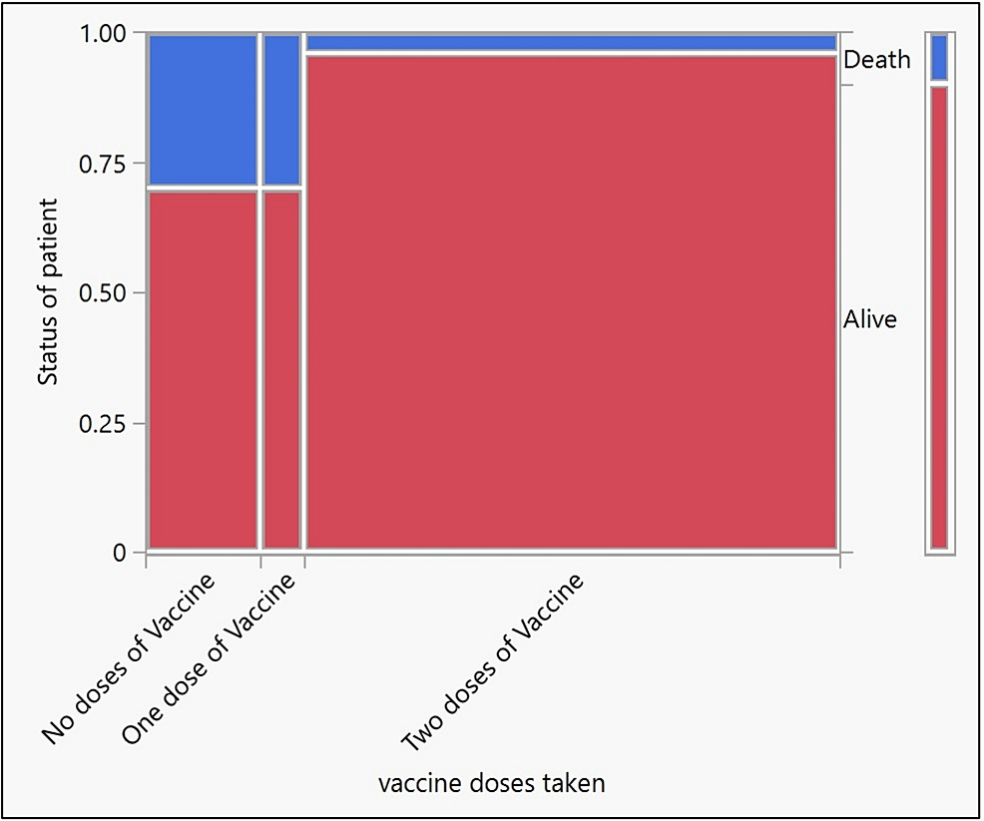
Mosaic plot showing the relationship between vaccination status and the outcome of confirmed Omicron cases

The demographic characteristics of 165 confirmed Omicron cases are shown in Table 2. Males (56.97%) were more affected than females (43.03%), with male to female ratio of 1.3: 1. The mean age of the study population was 36.51 (standard deviation = 17.02, 95% CI = 33.89 to 39.13). The age group 21 to 40 years were predominantly affected, followed by 41 to 60 years. Older age (greater than 60 years), an important risk factor for poor outcome, was present in 15/165 (9.1%) of cases. Figure 2 and Table 3 show the relationship between age and disease outcome. The presence of one or more comorbid conditions was seen in 15.15% of cases, diabetes mellitus (28%), hypertension (28%), obesity (16%), and interstitial heart disease (16%) were the most common conditions. Of the 165 cases, 81.82% were vaccinated with at least one dose of the COVID-19 vaccine (92.59% were vaccinated with two doses, and 7.41% were vaccinated with one dose of the vaccine), and 16.36% of the cases were unvaccinated. Figure 3 and Table 4 show the relationship between the vaccination status and the outcome of Omicron-confirmed cases.

**Table 2:** Demographic characteristics of 165 Omicron cases

Demographic characteristics	The number of cases survived (%)	Number of cases that died during their stay in the hospital (%)	Number of cases brought in dead to the hospital (%)	Total (%)	p-value
Age group (In years)					< 0.0001
0-20	14 (9.59%)	01 (8.33%)	01 (14.29%)	16 (9.70%)	
21-40	87 (59.59%)	01 (8.33%)	00 (00%)	88 (53.33%)	
41-60	39 (26.72%)	04 (33.33%)	03 (42.86%)	46 (27.88%)	
61-80	05 (3.42%)	06 (50%)	02 (28.57%)	13 (7.88%)	
>80	01 (0.68%)	00 (00%)	01 (14.29%)	02 (1.21%)	
Gender					
Male	82 (56.16%)	07 (58.33%)	05 (71.43%)	94 (56.97%)	
Female	64 (43.84%)	05 (41.67%)	02 (28.57%)	71 (43.03%)	
Area of Residence					
Rural	12 (8.22%)	03 (25%)	-	15 (9.09%)	
Urban	134 (91.78%)	09 (75%)	-	143 (86.67%)	
Data not available	00 (00%)	00 (00%)	07 (100%)	07 (4.24%)	
Marital Status					
Married	69 (47.26%)	10 (83.33%)	07 (100%)	86 (52.12%)	
Unmarried	77 (52.74%)	02 (16.67%)	00 (00%)	79 (47.88%)	
Socio-economic Status					
Average	38 (26.03%)	00 (00%)	00 (00%)	38 (23.03%)	
Poor	52 (35.62%)	12 (100%)	07 (100%)	71 (43.03%)	
Data not available	14 (9.59%)	00 (00%)	00 (00%)	14 (8.48%)	
Student	42 (28.77%)	00 (00%)	00 (00%)	42 (25.46%)	
Comorbidity Score					
No comorbid conditions present	133 (91.10%)	04 (33.33%)	03 (42.86%)	140 (84.85%)	
Presence of one or more condition	13 (8.90%)	08 (66.67%)	04 (57.14%)	25 (15.15%)	
Old age (Greater than 60 years- a risk factor)	06 (46.15%)	06 (75%)	03 (75%)	15 (60%)	
Diabetes Mellitus (DM)	02 (15.38%)	03 (37.50%)	02 (50%)	07 (28%)	
Hypertension (HTN)	01 (7.69%)	05 (62.50%)	01 (25%)	07 (28%)	
Obesity	04 (30.77%)	00 (00%)	00 (00%)	04 (16%)	
Interstitial Heart Disease (IHD)	01 (7.69%)	03 (37.50%)	00 (00%)	04 (16%)	
Liver Cirrhosis	00 (00%)	01 (12.50%)	01 (25%)	02 (08%)	
Haematological Disorder	00 (00%)	02 (25%)	00 (00%)	02 (08%)	
Pulmonary Tuberculosis	02 (15.38%)	00 (00%)	00 (00%)	02 (08%)	
Pulmonary venous disorder	00 (00%)	01 (12.50%)	00 (00%)	01 (04%)	
Chronic Kidney Disorder	00 (00%)	01 (12.50%)	00 (00%)	01 (04%)	
Cerebrovascular disorder	00 (00%)	01 (12.50%)	00 (00%)	01 (04%)	
Seizure disorder	00 (00%)	01 (12.50%)	00 (00%)	01 (04%)	
Psychiatric Illness	01 (7.69%)	00 (00%)	00 (00%)	01 (04%)	
Rheumatological Disorder	01 (7.69%)	00 (00%)	00 (00%)	01 (04%)	
Malignancy	00 (00%)	01 (12.50%)	00 (00%)	01 (04%)	
HIV	01 (7.69%)	00 (00%)	00 (00%)	01 (04%)	
Vaccination Status					< 0.0001
Vaccinated	127 (86.99%)	06 (50%)	02 (28.57%)	135 (81.82%)	
With one dose	07 (5.51%)	01 (16.67%)	02 (100%)	10 (7.41%)	
With two doses	120 (94.49%)	05 (83.33%)	00 (00%)	125 (92.59%)	
Not Vaccinated	19 (13.01%)	06 (50%)	02 (28.57%)	27 (16.36%)	
Data not available	00 (00%)	00 (0.00%)	02 (28.57%)	02 (1.21%)	
Not eligible for vaccination	00 (00%)	00 (00%)	01 (14.29%)	01 (0.61%)	

**Table 3:** Chi-square test results for goodness of fit showing the relationship between age and the outcome of disease

Test	ChiSquare (χ2)	Prob>ChiSq (p-value)
Likelihood Ratio	39.208	<0.0001
Pearson	55.154	<0.0001

**Table 4:** Chi-square test results for goodness of fit showing the relationship between vaccination status and the outcome of confirmed Omicron cases

Test	ChiSquare (χ2)	Prob>ChiSq (p-value)
Likelihood Ratio	17.43	0.0002
Pearson	21.24	<0.0001

Clinical profile of COVID-19 cases infected with the Omicron variant of SARS-CoV-2

**Figure 4:**
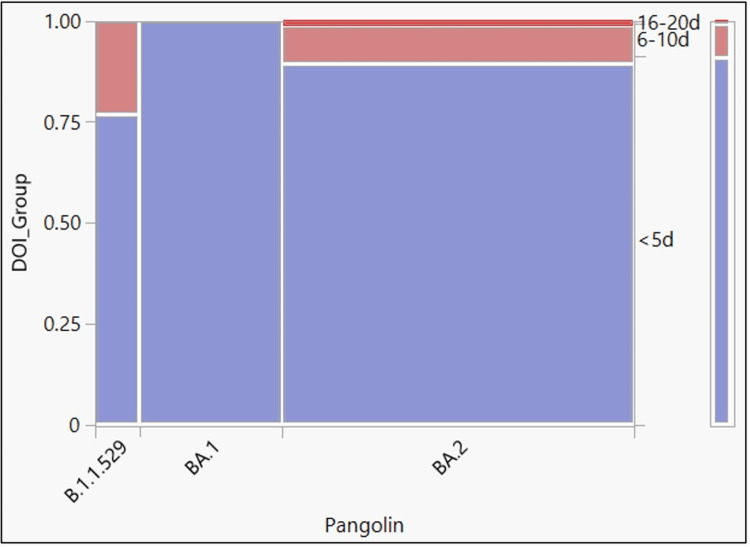
Mosaic plot showing the duration of illness versus Omicron variant Duration of Illness (DOI)

Out of 165 cases, the clinical characteristics of 158 (95.76%) Omicron cases could be recorded as the remaining seven cases were brought in dead (Table 5). Of these 158 cases, 86.71% had symptomatic disease, and 13.29% had an asymptomatic infection. Fever (67.72%) was the most common presenting symptom, followed by cough (40.51%), myalgia (35.44%), runny nose (22.78%), and headache (15.82%). The duration of illness was less than five days in 91.14% of cases, and only in 8.86% of cases the illness lasted for more than a week. Figure 4 and Table 6 show the relationship between the duration of illness to Omicron variants. The mean duration of illness on admission was 2.69 days (standard deviation = 2.12, 95% CI = 2.36 to 3.02). Table 7 describes the biochemical and hematological parameters of the hospitalized Omicron cases.

**Table 5:** Clinical characteristics of 158 Omicron cases National Early Warning Score (NEWS)

Clinical characteristics	Number of cases survived (%)	Number of cases that died during their stay in the hospital (%)	Total (%)	p-value
Symptom status				
Asymptomatic	21 (14.38%)	00 (00%)	21 (13.29%)	
Symptomatic	125 (85.62%)	12 (100%)	137 (86.71%)	
Initial presenting symptoms				
Fever	103 (70.55%)	04 (33.33%)	107 (67.72%)	
Anosmia	00 (00%)	07 (58.33%)	07 (4.43%)	
Ageusia	00 (00%)	08 (66.67%)	08 (5.06%)	
Rhinorrhoea	36 (24.66%)	00 (00%)	36 (22.78%)	
Cough	60 (41.10%)	04 (33.33%)	64 (40.51%)	
Breathlessness	06 (4.11%)	08 (66.67%)	14 (8.86%)	
Headache	25 (17.12%)	00 (00%)	25 (15.82%)	
Myalgia	56 (38.36%)	00 (00%)	56 (35.44%)	
Vomiting	03 (2.05%)	00 (00%)	03 (1.90%)	
Fatigue/ Weakness	15 (10.27%)	02 (16.67%)	17 (10.76%)	
Abdominal Pain	00 (00%)	01 (8.33%)	01 (0.63%)	
Duration of Illness				
Less than 5 days	139 (95.21%)	05 (41.67%)	144 (91.14%)	
More than 6 days	07 (4.79%)	07 (58.33%)	14 (8.86%)	
NEWS score				0.3072
1 to 4	139 (95.21%)	02 (16.67%)	141 (89.24%)	
5 to 8	07 (4.79%)	07 (58.33%)	14 (8.86%)	
More than 9	00 (00%)	03 (25%)	03 (1.90%)	
Number of Organ systems involvement				< 0.0001
No Organ system involvement	102(69.86%)	00 (00%)	102 (64.56%)	
The involvement of one or more organ systems	44 (30.14%)	12 (100%)	56 (35.44%)	
Single-system involvement	25 (56.82%)	00 (00%)	25 (44.64%)	
Two-system involvement	13 (29.55%)	00 (00%)	13 (23.21%)	
Three or more systems involvement	06 (13.64%)	12 (100%)	18 (32.14%)	
Chest X-ray findings in admitted cases (n=82)				
Normal X-ray findings	67 (100%)	10 (66.67%)	77 (93.90%)	
Abnormal findings	00 (00%)	05 (33.33%)	05 (6.10%)	
Pulmonary infiltrates	00 (00%)	02 (40%)	02 (40%)	
Pulmonary oedema	00 (00%)	03 (60%)	03 (60%)	
Type of treatment given				
Conservative treatment	146 (100%)	00 (00%)	146 (92.41%)	
In-patient department	70 (47.95%)	00 (00%)	70 (47.95%)	
Out-patient department	76 (52.05%)	00 (00%)	76 (52.05%)	
Need for oxygen therapy	00 (00%)	12 (100%)	12 (7.59%)	
Duration of hospital stay among cases who were given standard care (n=82)				
Less than 24 hours	00 (00%)	01 (8.33%)	01 (1.22%)	
1 day to 2 days	07 (10%)	02 (16.67%)	09 (10.98%)	
3 days to 4 days	25 (35.71%)	00 (00%)	25 (30.49%)	
5 days to 9 days	36 (51.43%)	04 (33.33%)	40 (48.78%)	
Greater than 10 days	02 (2.86%)	05 (41.67%)	07 (8.53%)	
Duration of non-invasive ventilation among cases requiring oxygen therapy (n=12)				
Less than 24 hours	00 (00%)	04 (33.33%)	04 (33.33%)	
1 day to 2 days	00 (00%)	05 (41.67%)	05 (41.67%)	
Greater than 3 days	00 (00%)	03 (25%)	03 (25%)	
Duration of invasive ventilation among cases requiring oxygen therapy (n=12)				
Less than 6 hours	00 (00%)	02 (16.67%)	02 (16.67%)	
6 hours to 12 hours	00 (00%)	05 (41.67%)	05 (41.67%)	
12 hours to less than 24 hours	00 (00%)	01 (8.33%)	01 (8.33%)	
1 day to 2 days	00 (00%)	02 (16.67%)	02 (16.67%)	
3 days to 7 days	00 (00%)	02 (16.67%)	02 (16.67%)	

**Table 6:** Chi-square test results for goodness of fit showing the relationship between duration of illness versus Omicron variant

Test	ChiSquare (χ2)	Prob>ChiSq (p-value)
Likelihood Ratio	11.046	0.026
Pearson	8.44	0.077

**Table 7:** Laboratory parameters of hospitalized Omicron cases Note: The comparison of laboratory parameters between survived and dead cases could not be calculated statistically as the numbers were low.

Laboratory Parameters	Reference range	Number of cases	Mean value	Standard deviation	Upper and lower 95% CI
Biochemical Parameters					
Liver Function Tests					
Total Bilirubin (n= 139)			0.60	0.47	0.68 – 0.52
Low		00			
Normal	0.3 – 1.0 mg/dL	128			
High		11			
Direct Bilirubin (n=123)			0.31	0.23	0.35 – 0.27
Low		00			
Normal	– 0.3 mg/dL	99			
High		24			
Serum Glutamate Pyruvate Transaminase (SGPT) (n= 134)			34.19	30.08	39.33 – 29.05
Low		00			
Normal	0 – 45 IU/L	121			
High		13			
Serum Glutamic-Oxaloacetic Transaminase (SGOT) (n= 135)			36.68	41.43	43.73 – 29.63
Low		00			
Normal	0 – 35 IU/L	122			
High		13			
Alkaline Phosphatase (ALP) (n= 98)			66.93	35.35	74.01 – 59.84
Low		18			
Normal	30-120 IU/L	79			
High		01			
Total Proteins (n= 112)			6.97	0.73	7.10 – 6.83
Low		06			
Normal	3.0 – 8.0 g/dL	106			
High		00			
Serum Albumin (n= 120)			3.88	0.70	4.01 – 3.76
Low		24			
Normal	40 – 60 g/L	95			
High		01			
Creatine Kinase-Myocardial Band (CK-MB) (n= 42)			41.59	89.62	69.51 – 13.66
Low		02			
Normal	5 – 25 IU/L	20			
High		20			
Serum Ferritin (n= 54)			246.34	326.73	335.52 – 157.16
Low		06			
Normal	Male: 30.3 to 565.7 ng/mL Female: 14.7 to 205.1 ng/mL	34			
High		14			
Serum Lactate Dehydrogenase (LDH) (n= 78)			530.73	754.58	700.86 – 360.60
Low		00			
Normal	Male: 135 – 225 IU/L Female: 135 – 214 IU/L	15			
High		63			
Interleukin-6 (IL-6) (n= 21)			956.58	1722.50	1740.65 – 172.51
Low		00			
Normal	0 – 7 pg/mL	07			
High		14			
D-Dimer (n= 37)			1.59	2.65	2.47 – 0.71
Low		00			
Normal	< 0.50	14			
High		24			
Renal Function Tests					
Serum Creatinine (n= 135)			1.27	0.87	1.42 – 1.13
Low		00			
Normal	Male: 0.74 – 1.35 mg/dL Female: 0.59 – 1.04 mg/dL	130			
High		05			
Serum Urea (n= 124)			34.68	38.94	41.60 – 27.76
Low		00			
Normal	Male: 6 – 21 mg/dL Female: 8 – 24 mg/dL	70			
High		54			
Serum Uric Acid (n= 101)			5.48	3.08	6.09 – 4.87
Low		28			
Normal	3.5 – 7.2 mg/dL	64			
High		09			
Serum Sodium (n= 136)			135.92	6.58	137.03- 134.80
Low		62			
Normal	135 – 145 mmol/L	67			
High		07			
Serum Potassium (n= 136)			3.84	0.53	3.93 – 3.75
Low		35			
Normal	3.5 – 5.2 mmol/L	98			
High		03			
Hematological Parameters					
Haemoglobin (n = 137)			13.08	2.35	13.48 – 12.68
Low		49			
Normal	Male: 13.5 – 17.5 g/dL Female: 12.0 – 16.0 g/dL	88			
High		00			
Red Blood Cell (RBC) Count (n= 81)			4.97	0.90	5.17 – 4.77
Low		04			
Normal	Male: 4.3 – 5.9 million/mm^3^ Female: 3.5 – 5.5 million/ mm^3^	68			
High		09			
White Blood Cell (WBC) Count (n= 134)			5.99	3.52	6.60 – 5.39
Low		07			
Normal	4,500 – 11,000/mm^3^	117			
High		10			
Neutrophil to Lymphocyte Ratio (n= 52)			2.33	0.18	2.39 – 2.29
Low		00			
Normal	0.78 – 3.53	52			
High		00			
Platelet Count (n= 140)			246.25	72.19	258 – 234.19
Low		07			
Normal	1,50,000 – 4,00,000/mm^3^	133			
High		00			
Fasting Blood Sugar Level (n= 31)			129.26	41.74	144.57 – 113.95
Low		00			
Normal	70 – 99 mg/dL	28			
High		03			

**Figure 5:**
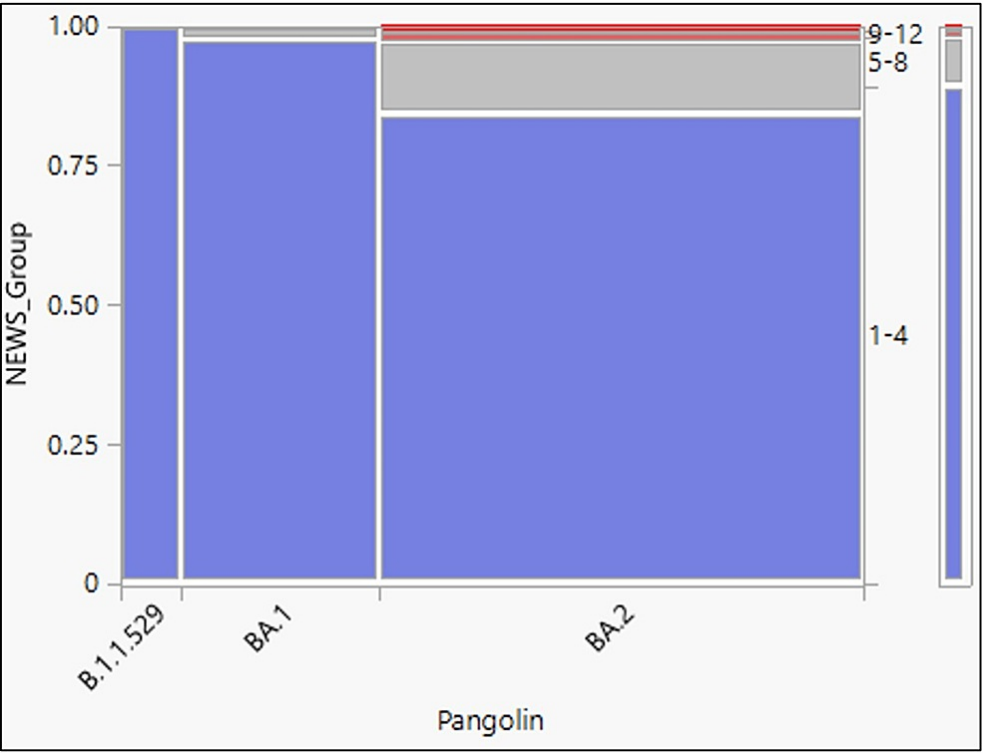
Mosaic plot showing National Early Warning Score (NEWS) versus Omicron variant NEWS - National Early Warning Score

Based on the National Early Warning Score (NEWS), patients were classified as having low scores (NEWS 1-4), medium scores (NEWS of 5-6), and high scores (NEWS > 7). Of the 158 cases, 89.24% had a score of 1-4 (low score), and 8.86% had a score between 5-8. Figure 5 and Table 8 show an analysis of the NEWS versus the Omicron variant. The mean NEWS was 2.71 (standard deviation = 2.19, 95% CI = 2.36 to 3.05).

**Table 8:** Chi-square test results for goodness of fit showing the relationship between National Early Warning Score (NEWS) versus Omicron variant

Test	Chi-Square (χ2)	Prob>ChiSq (p-value)
Likelihood Ratio	9.887	0.1295
Pearson	7.150	0.3072

No systemic involvement was seen in 64.56% of the cases, while in 35.44% of cases, one or more organ systems were involved. The gastrointestinal and hepatobiliary system was most commonly affected (55.4%), followed by the cardiac system (50%), central nervous system (10.7%), renal system (8.93%), and hematological system (8.93%). Sepsis was seen in 17.9% of cases. For 82 cases, chest X-ray findings were available, of which 77 (93.90%) cases had normal X-Ray findings, whereas five (6.10%) cases had abnormal findings. Of these five cases, two (40%) had pulmonary infiltrates, and three (60%) had evidence of pulmonary oedema. These abnormal findings were seen in cases that succumbed to the disease.

Out of 158 cases, 146 (92.41%) cases recovered with conservative treatment, and 12 (7.59%) cases required additional oxygen therapy. Among those requiring oxygen therapy, 41.67% of cases required invasive ventilation for six to 12 hours. Of the 82 cases admitted, 48.78% were hospitalized for five to nine days.

## Discussion

The most recently discovered novel SARS-CoV-2 variant, the Omicron variant, has a much higher effective reproduction number than the Delta variant (3.6 to 4.2 times) and a shorter incubation period. Among all the VOCs detected to date, the Omicron variant possesses the highest number of mutations in its genome, with 32 mutations in its spike protein. Mutations in the spike protein like Q493R, T478K, and N501Y bind to the host cell's angiotensin-converting enzyme 2 (ACE2) receptors with higher affinity than the wild type and other VOCs. Similarly, mutations like H655Y, N679K, and P681H increase cleavage activity by host furin. Such mutations in the spike protein led to increased infectivity and transmissibility [8,4].

The present study suggests that Omicron cases suffered from mild disease with 91.14% of cases having the illness for less than five days. Around 89.24% of cases had a NEWS between 1 to 4 suggesting a good prognosis. More than 90% of cases showed no lung involvement and recovered with conservative treatment. Death due to Omicron infection was seen in 11.5% of cases with most deaths concentrated in the population 60 years and older and those with comorbidities. Our results are consistent with earlier studies in South Africa that suggested a significant reduction in pathogenicity, severity, and mortality rates of the disease during the Omicron wave [9-11]. Similar findings have also been reported in countries such as the United States, France, South Korea, Japan [12-15], and China [8] where both the vaccination coverage and population infection were quite high. However, in the present study, the number of cases was small. As a result, the numbers under different subgroups were even smaller. With increasing numbers of cases presenting with no symptoms or requiring no hospital admission, clinical data in such cases became less available.

Transmembrane serine proteases 2 (TMPRSS2) facilitate viral entry into the host cell. The wild-type variant and other VOCs efficiently utilize this cell surface fusion pathway to enter the host cell. However, in the case of Omicron, the variant is inefficient in using this pathway. Studies indicate that Omicron depends on cathepsins and enters the host cell primarily through the endosomal pathway. The co-expression of ACE2 and cathepsins are abundant in the upper respiratory tract, explaining the increased replication of Omicron in bronchi than in the lungs. Therefore, in Omicron infection, there is reduced lung pathology and diminished pro-inflammatory responses [16]. This property probably explains the reduced disease severity, the reduced need for oxygen and hospital admission, and better survival outcomes in the Omicron cases compared to other VOCs. Our results are consistent with these findings, wherein 93.90% of cases had no lung involvement and 92.41% recovered with conservative treatment without any additional oxygen requirement.

Since the Omicron variant has acquired numerous mutations in its spike protein, particularly in its receptor-binding domain (RBD), this has increased the likelihood of reduced efficacy of the neutralizing antibodies on Omicron. Various studies have shown that Omicron partially or completely escapes neutralization by antibodies present in convalescent sera [17,18] and vaccinated individuals [19-21], thus suggesting evasion of natural and vaccine-induced immunity. Also, monoclonal antibodies, either clinically approved or in development for treatment, have completely failed to neutralize Omicron [22,23,18]. Mutations like E484A and K417N dodge the neutralizing antibodies, eventually resulting in an increased ability of immune escape [4]. Therefore, this probably explains the increased frequency of breakthrough infections and reinfection incidents during the Omicron wave. Besides the antibody-mediated immunity, studies have shown that T-cell responses to the Omicron variant are primarily preserved, and T-cell escape is minimal compared to other VOCs. Also, the booster vaccine doses enhance T-cell responses [24]. In the present study, the vaccination rate in the population under investigation was good, and it could have potentially contributed to the reduced severity of the disease. Therefore, good vaccination coverage is desirable for effective protection against severe disease [25].

The present study could not compare the differences in laboratory parameters between the survived and deceased cases due to the unavailability of investigation results for brought-in dead cases and a low number of in-hospital deaths. This fact limited our ability to draw definitive conclusions about the relationship between these laboratory parameters and the survival outcomes.

## Conclusions

To conclude, the present study described the baseline characteristics of laboratory-confirmed Omicron variant cases in Pune, Maharashtra. Our analysis indicates Omicron causes a mild disease, with a reduced need for hospital admission, and a lower need for oxygen therapy. The overall lowered disease severity reflects the contribution of attenuated intrinsic virulence of the virus as well as the impact of prior immunity due to natural infection and/or vaccination. Vaccine breakthrough infections were commonly seen. Additional studies will continue to be essential in understanding the evolution of current and future variants in populations with a diverse immunological landscape.
